# The relationship between platelet distribution width and new-onset cardiovascular disease events in patients with peritoneal dialysis

**DOI:** 10.1080/0886022X.2022.2130802

**Published:** 2022-10-25

**Authors:** Ning Su, Xingming Tang, Xiaojiang Zhan, Xiaoyang Wang, Fenfen Peng, Yueqiang Wen, Xiaoran Feng, Qian Zhou, Qinqin Wang, Xingyu Chen, Yuanyuan Yang, Sijia Shang

**Affiliations:** a Department of Nephrology, The Sixth Affiliated Hospital, Sun Yat-sen University, Guangzhou, China; b Department of Hematology, The Sixth Affiliated Hospital, Sun Yat-sen University, Guangzhou, China; c Department of Nephrology, DongGuan SongShan Lake Tungwah Hospital, DongGuan, China; d Department of Nephrology, The First Affiliated Hospital, Nanchang University, Nanchang, China; e Department of Nephrology, The First Affiliated Hospital of Zhengzhou University, Zhengzhou University, Zhengzhou, China; f Department of Nephrology, Zhujiang Hospital, Southern Medical University, Guangzhou, China; g Department of Nephrology, The Second Affiliated Hospital, Guangzhou Medical University, Guangzhou, China; h Department of Nephrology, Jiujiang No. 1 People’s Hospital, Jiujiang, China; i Department of Medical Statistics, Clinical Trials Unit, The First Affiliated Hospital, Sun Yat-sen University, Guangzhou, China

**Keywords:** Platelet distribution width, platelet counts, dialysis, cardiovascular disease events

## Abstract

**Objectives:**

The global mortality rate from chronic kidney disease (CKD) has increased over the past two decades. Typically, peritoneal dialysis (PD) remains a useful alternative treatment for end-stage renal disease. Cardiovascular disease (CVD) is the main complication in PD patients. In terms of prognosis, it is reported that platelet distribution width (PDW) can predict adverse CVD events. However, the relationship between PDW and new-onset CVD in PD patients is not clear. This study aimed to explore the relationship between PDW and new-onset CVD in PD patients.

**Methods:**

This was a retrospective cohort study, from 4 July 2005 to 31 December 2019, and a total of 1557 patients were recruited. PDW was respectively categorized into two groups: PDW ≤13.2 fL and PDW >13.2 fL. The primary outcome was a new-onset CVD event. Cox proportional hazards models were performed to assess the hazard ratio (HR). Receiver-operating characteristic (ROC) curves were applied to evaluate the predictive accuracy of the PDW on CVD events.

**Results:**

During follow-up, 114 new-onset CVD events were recorded. Cox proportional hazards models showed a higher risk of CVD events in patients with high PDW (HR = 1.862 95%CI 1.205–2.877, *p* = 0.005). Kaplan–Meier cumulative incidence curves showed the risk of the first occurrence of CVD events was greater in the high PDW group (*p* = 0.006).

**Conclusions:**

High PDW is associated with new-onset cardiovascular disease events in PD patients.

## Introduction

Chronic kidney disease (CKD) has become a global health problem [1]. The global mortality rate from CKD increased by 41.5% between 1990 and 2017. CKD has become a leading cause of global morbidity and mortality [2]. In terms of treatment, the number of patients receiving dialysis exceeded 2.5 million and was projected to achieve 5.4 million by 2030, statistically [3]. Thereinto, peritoneal dialysis (PD) remains an economical, convenient, and effective alternative treatment for end-stage renal disease [4]. It is reported that CKD is associated with an enhanced risk of all types of cardiovascular disease (CVD) [5]. Meanwhile, CVD is the main cause of death among patients undergoing maintenance dialysis. A collaborative study on dialysis showed that CVD morbidity in PD patients was 58.9%, whereas CVD morbidity in HD patients was 56.3% [6].

Inflammation and thrombosis play key roles in the occurrence of CVD in CKD patients [7–9]. The activation of inflammatory cells and the release of inflammatory cytokines are independent risk factors for CVD in CKD patients (including PD patients) [10–14]. In the animal models of CKD, it is also found that excessive activation of the inflammation is associated with CVD events [15]. Meanwhile, it is found that oxidative stress and apoptosis were associated with endothelial cell culture by extracting serum from CKD rats [16]. Similarly, thrombosis is found to be involved in the development of CVD in animal models and populations of CKD [17–20].

Platelets are not only effectors of thrombosis, but also actively involved in inflammation. Activated platelets release proinflammatory factors to amplify inflammation [21]. Meanwhile, the activated platelets induce endothelium to release pro-inflammatory compounds such as IL-1β and CD40L, which recruit the circulating white blood cells to adherent endothelium and form lipid plaques [22]. Furthermore, activated platelets usually manifest as their change in volume, which is reflected in a change in platelet volume indices [23]. Platelet distribution width (PDW), defined as the distribution width (femtoliter, fL) at 20% of the total height of the platelet size distribution curve, is one of the most validated and prominent of platelet volume indices [23,24]. In recent years, there have been many reports on PDW. Elevated PDW is presumed to be associated with atherosclerosis, coronary artery disease, stroke and inflammatory disease [25–27]. A cohort study suggests that lower PDW values are significantly related to lower risks of CVD [28]. Moreover, one study has shown that lower platelet counts (PLT) and PDW can predict CVD events in CKD patients without dialysis [29]. However, there is no research on the interaction between PDW and new-onset CVD in patients undergoing PD. Thus, this study aims to investigate the relationship between PDW and CVD events in PD patients.

## Methods

### Patients

**Figure 1.**
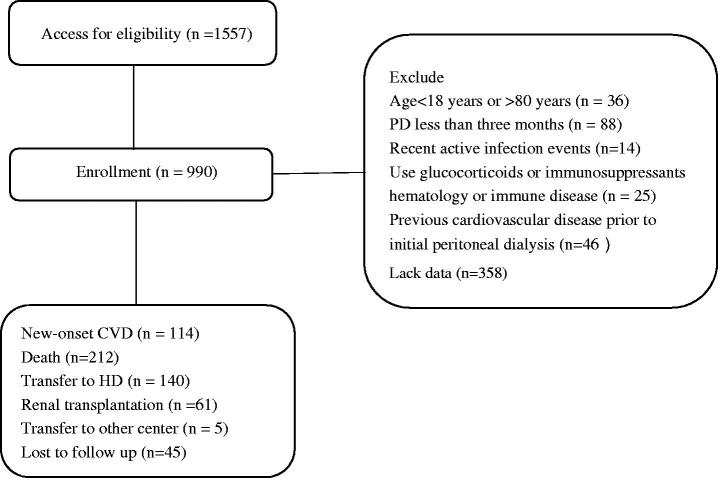
Study flow, including patient enrollment and outcomes. CVD: cardiovascular disease; HD: hemodialysis; PD: peritoneal dialysis.

This was a retrospective, observational cohort study. A total of 1557 patients were recruited from four PD centers from 4 July 2005 to 30 December 2019. The reasons that 567 patients were excluded from this study were as follows: age < 18 years or >80 years, PD was maintained for less than three months, recent active infection events, using glucocorticoids or immunosuppressants or suffering from the immune or hematology disease, suffering from previous CVD before initial PD, and lack of data. A total of 990 PD patients were eventually enrolled in this study. The flow chart is shown in Figure 1. Written informed consent was required from all patients. The study was in line with the ethical principles of the Helsinki declaration, and approved by the Ethics Committee of the Sixth Affiliated Hospital of Sun Yet-Sen University (No. 2021SLYEC-177)

### Outcomes

The primary outcome was the new-onset CVD events during follow-up. New-onset CVD events were defined as the first occurrence of any of the following conditions after the onset of PD: coronary heart disease, coronary atherosclerotic heart disease, acute myocardial infarction, cardiac arrest, cerebrovascular accident, stroke, and congestive heart failure. All patients were followed up until new-onset CVD, death, transferring to hemodialysis therapy, transferring to renal transplantation, transferring to other centers, loss to follow-up, or censoring on 1 April 2020.

### Clinical data

Baseline demographic data including age, gender, body mass index (BMI), a history of diabetes and hypertension, and laboratory parameters including PLT, PDW, neutrophil, lymphocyte, hemoglobin, serum albumin, total Kt/V, 24 h urine output, residual renal function (RRF), total cholesterol, triglycerides, serum uric acid, serum creatinine, serum calcium, and serum phosphorus were obtained. Patients with a record of current use of insulin or oral hypoglycemic drugs and/or a clinical diagnosis of type 1 or type 2 diabetes were considered to have diabetes. Hypertension was recorded if the patient took antihypertensive medications or had two separate measurements of blood pressure ≥140/90 mmHg. RRF, defined as glomerular filtrate that has escaped tubular reabsorption, [30], was quantified as mean values of creatinine clearance and adjusted for body surface area.

All parameters were measured through standard methods in the clinical laboratory. BMIwas calculated as weight (kg) divided by height (m) squared. Residual renal function and total Kt/V were calculated using PD Adequest software 2.0 (Baxter Healthcare Ltd). RRF (mL/min/1.73 m^2^) was measured from mean values of creatinine clearance and adjusted for body surface area.

### Statistical analysis

According to the median, high PDW was defined as PDW > 13.2 fL. For a further study on the relationship between platelets and CVD events in PD patients, participants were also divided into two groups of PLT, and high PLT was defined as PLT > 163 (10^9^/L). Baseline characteristics were presented as percentages (%) for categorical variables, mean ± standard deviations for normally distributed variables, and median (25th–75th percentile) for non-normally distributed variables. The Chi-square test and Mann–Whitney test were used to test for differences in categorical or continuous factors among the groups. Univariable Cox regression was used to examine the association between patients’ characteristics and new-onset CVD events. Survival curves were calculated using the Kaplan–Meier method. Four Cox proportional hazard models were utilized to evaluate the relationship between the two PLT indices and the risk of new-onset CVD. Receiver-operating characteristic (ROC) curves were applied to evaluate the predictive accuracy of the two platelet indices on CVD events. Parameters with the area under ROC (AUC) >0.5 were treated as potentially significant prognostic indicators of new-onset CVD events. Interactions between subgroup variables including sex, age, history of hypertension, and history of diabetes, and the PDW group were investigated by performing a formal interaction test. Forest plot was utilized to represent the relationship between PDW and new-onset CVD events in different groups.

Statistical analysis was completed by SPSS 25.0, whereas survival analysis, ROC curve, and forest plot were performed with the use of GraphpadPrism 8.0. All tests were performed bilaterally, and *p* < 0.05 was considered to be statistically significant.

## Results

### Baseline characteristics

**Figure 2.**
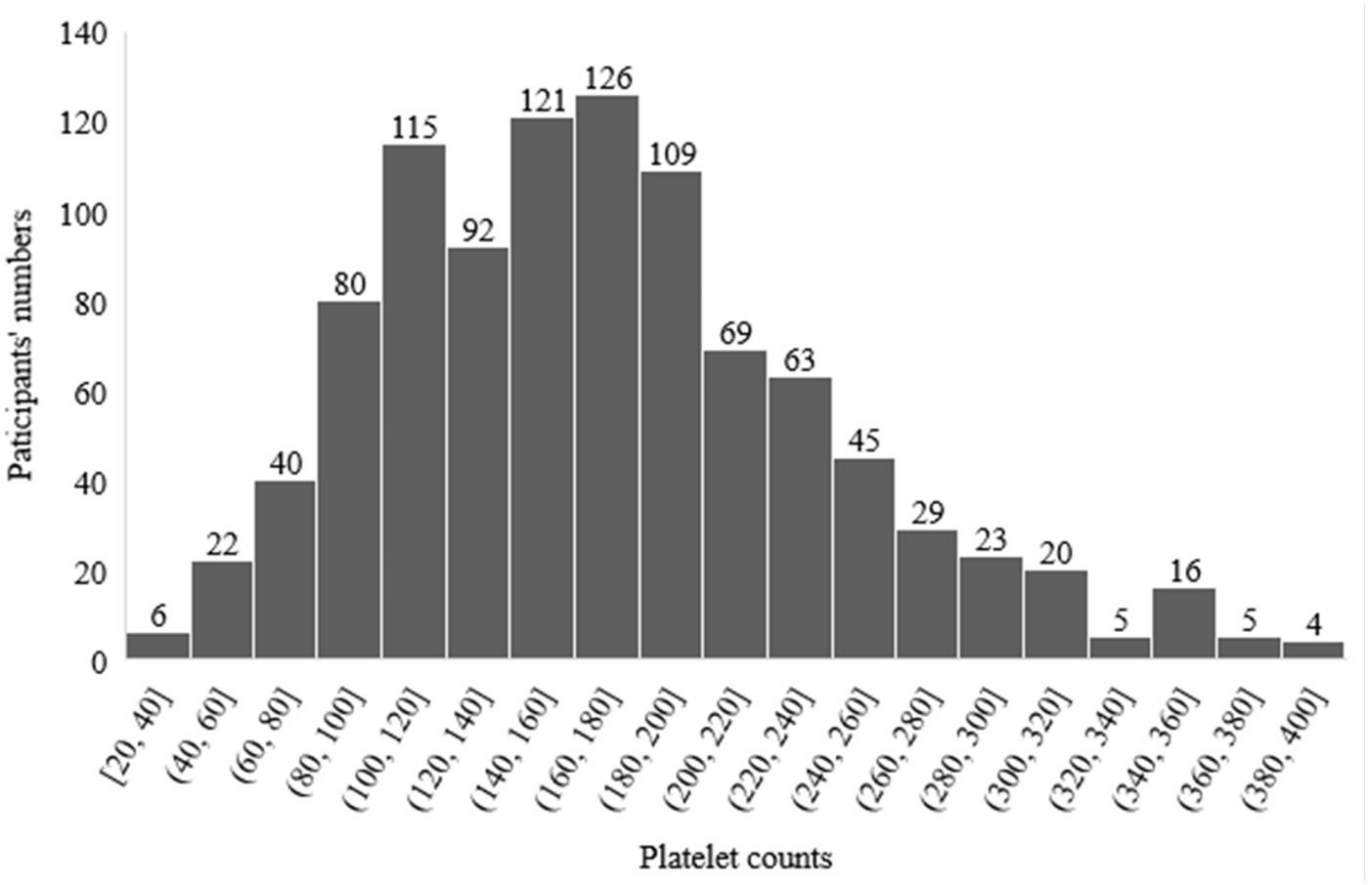
Distribution of platelet counts.

**Figure 3.**
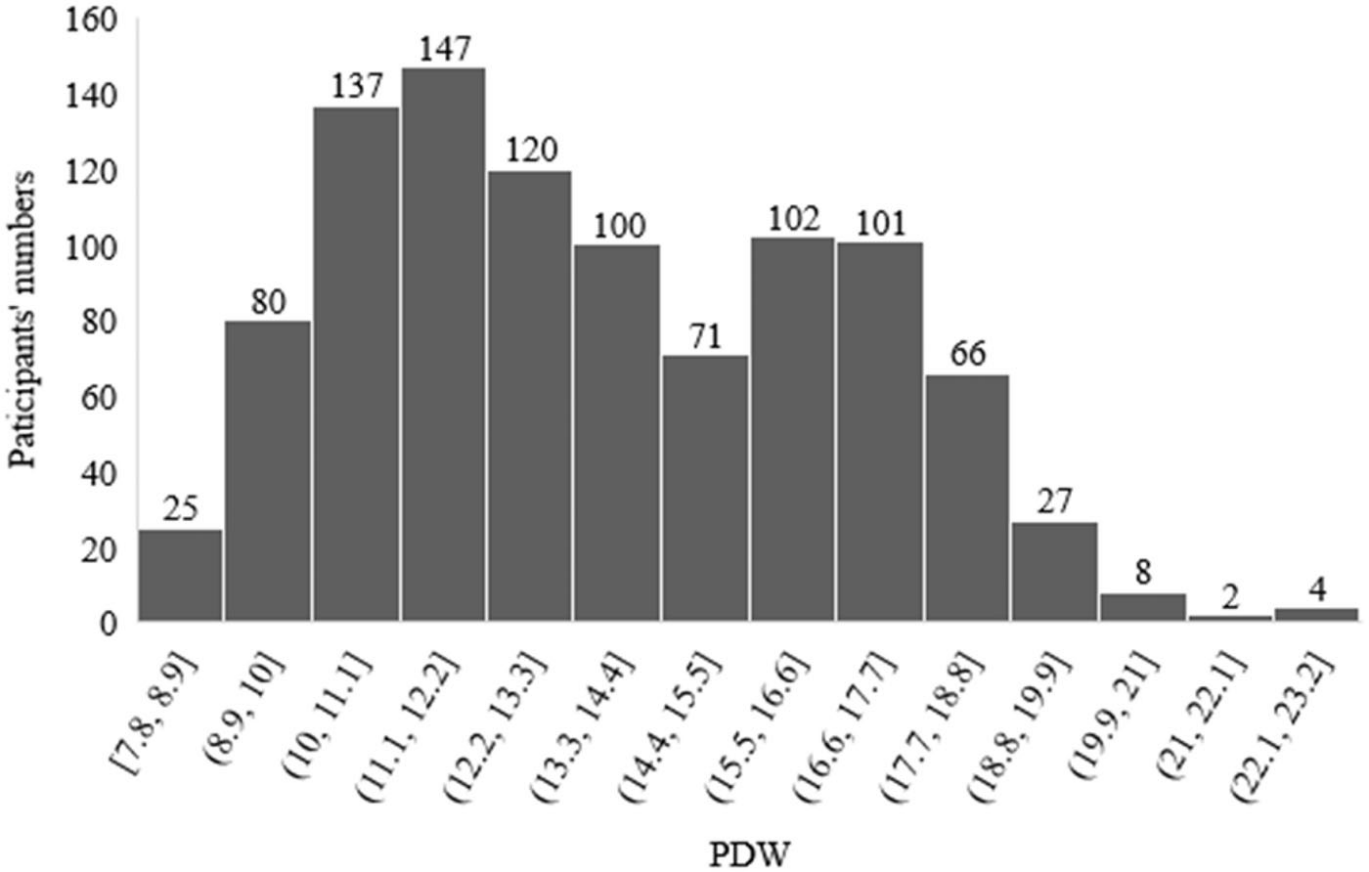
Distribution of PDW. PDW: platelet distribution width.

A total of 990 PD patients were eventually enrolled in this study, following up to death (*n* = 212), conversion to hemodialysis (*n* = 140), conversion to renal transplantation (*n* = 61), transferring to other centers (*n* = 5), lost to follow-up (*n* = 45). With a median follow-up of 63.5 months, 114 new-onset CVD were recorded (Figure 1). The distribution of patients according to PDW level and PLT level is shown in Figures 2 and 3. Comparisons of baseline demographic and clinical characteristics of the cohort study among groups are, respectively, given in Tables 1 and 2. Patients in the high PLT group had a higher rate of diabetes and higher levels of BMI, neutrophil, lymphocyte, hemoglobin, cholesterol, triglycerides, RRF, and calcium, but lower serum urea nitrogen, creatinine, albumin, and phosphorus. Meanwhile, patients in high PDW group had a higher rate of females and higher levels of serum urea nitrogen, uric acid, creatinine, albumin, and phosphorus, but lower dialysis vintage, cholesterol, RRF, creatinine, and a lower rate of hypertension.

**Table 1.** Baseline Characteristics of patients according to groups of PDW.

Variables	Total (*n* = 990)	PD*W* ≤ 13.2fL （*n* = 498）	PD*W* > 13.2fL （*n* = 492）	*p*-Value
Dialysis vintage (months)	39.00 (17.75–63.00)	42.00 (19.75–64.00)	35.00 (15.00–61.00)	0.009
Age (years)	48.00 (38.00–59.00）	48.00 (38.00–58.00）	48.00 (38.00–59.00）	0.719
Male sex (%)	574 (58.0)	308 (61.8)	266 (54.1)	0.013
BMI (kg/m^2^)	21.51 (19.62–24.10)	21.34 (19.62–24.08)	21.67 (19.56–24.22)	0.046
History of diabetes (%)	171 (17.3)	87 (17.5)	84 (17.1)	0.869
History of hypertension (%)	716 (72.3)	376 (75.5)	340 (69.1)	0.025
Neutrophil (10^9^/L)	3.90 (3.00–5.18)	3.92 (2.97–5.28)	3.90 (3.00–5.10)	0.739
Lymphocyte (10^9^/L)	1.14 (0.87–1.43)	1.14 (0.87–1.43)	1.10 (0.87–1.43)	0.170
Hemoglobin (g/L)	79.00 (68.00–91.00)	79.00 (68.00–91.00)	77.00 (68.00–90.00)	0.938
Total cholesterol (mmol/L)	4.10 (3.37–4.89)	4.17 (3.40–4.92)	4.04 (3.33–4.82)	0.031
Total triglycerides (mmol/L)	1.28 (0.89–1.78)	1.27 (0.87–1.83)	1.29 (0.93–1.73)	0.893
Serum urea nitrogen (mmol/L)	22.00 (17.10–28.80)	21.35 (16.10–28.20)	23.10 (17.70–30.00)	<0.001
Uric acid (μmol/L)	434.00 (345.00–534.00)	424.00 (338.25–524.75)	458.00 (352.00–540.00)	0.001
Total Kt/V	2.16 (1.67–2.71)	2.16 (1.67–2.69)	2.17 (1.67–2.73)	0.686
24h urine output (ml)	800.00 (500.00–1200.00)	800.00 (500.00–1200.00)	800.00 (500.00–1100.00)	0.483
RRF (ml/min/1.73 m^2^)	3.11 (1.72–5.36)	2.90 (1.64–5.07)	3.31 (1.89–5.67)	0.049
Serum creatinine (µmol/L)	698.00 (557.60–874.00)	702.50 (566.32–861.48)	693.00 (536.10–884.20)	0.022
Serum albumin (g/L)	36.00 (32.40–39.30)	35.95 (32.00–39.18）	36.1 (32.90–39.50）	0.011
Serum calcium (mmol/L)	2.01 (1.82–2.16)	2.03 (1.82–2.16)	1.98 (1.81–2.16)	0.185
Serum phosphorus (mmol/L)	1.82 (1.50–2.12)	1.82 (1.50–2.07)	1.85 (1.48–2.20)	0.003

*Note*: Normal reference range: serum albumin: 40.00–55.00g/L; serum calcium: 2.08–2.80 mmol/L ; serum phosphorus: 0.83–1.96 mmol/L. RRF was calculated as (urine creatinine [μmol/l] × urine volume [ml])/(plasma creatinine [μmol/l] × minutes) and corrected for body surface area.

PDW: platelet distribution width; BMI: body mass index; RRF: residual renal function.

**Table 2.** Baseline characteristics of patients according to groups of PLT.

Variables	Total (*n* = 990)	PL*T* ≤ 163.00 (10^9^/L) （*n* = 498）	PL*T* > 163.00 (10^9^/L) （*n* = 492）	*p*-Value
Dialysis vintage (months)	39.00 (17.75–63.00)	39.00 (18.00–64.00)	39.00 (17.00–61.75)	0.188
Age (years)	48.00 (38.00–59.00）	47.00 (37.00–58.00）	49.00 (40.00–60.00）	0.105
Male sex (%)	574 (58.0)	284 (57.0)	290 (58.9)	0.542
BMI (kg/m^2^)	21.51 (19.62–24.10)	21.11 (18.90–23.55)	22.04 (20.08–24.46)	0.001
History of diabetes (%)	171 (17.3)	69 (13.9)	102 (20.7)	0.004
History of hypertension (%)	716 (72.3)	357 (71.7)	359 (73.0)	0.652
Neutrophil (10^9^/L)	3.90 (3.00–5.18)	3.55 (2.70–4.83)	4.40 (3.39–5.61)	<0.001
Lymphocyte (10^9^/L)	1.14 (0.87–1.43)	1.03 (0.79–1.30)	1.21 (0.97–1.54)	<0.001
Hemoglobin (g/L)	79.00 (68.00–91.00)	75.00 (66.00–86.00)	83.00 (72.00–94.00)	<0.001
Total cholesterol (mmol/L)	4.10 (3.37–4.89)	3.94 (3.23–4.70)	4.30 (3.56–5.03)	<0.001
Total triglycerides (mmol/L)	1.28 (0.89–1.78)	1.20 (0.81–1.63)	1.39 (0.96–1.96)	<0.001
Serum urea nitrogen (mmol/L)	22.00 (17.10–28.80)	23.60 (17.80–30.00)	20.55 (16.20–27.67)	<0.001
Uric acid (μmol/L)	434.00 (345.00–534.00)	438.00 (320.00–531.00)	429.00 (346.00–535.50)	0.530
Total Kt/V	2.16 (1.67–2.71)	2.15 (1.63–2.72)	2.18 (1.70–2.70)	0.433
24h urine output (ml)	800.00 (500.00–1200.00)	800.00 (500.00–1100.00)	800.00 (500.00–1207.00)	0.008
RRF (ml/min/1.73 m^2^)	3.11 (1.72–5.36)	2.88 (1.53–4.92)	3.36 (1.92–5.65)	0.005
Serum creatinine (µmol/L)	698.00 (557.60–874.00)	761.00 (589.50–988.93)	695.00 (559.75–872.00)	<0.001
Albumin (g/L)	36.00 (32.40–39.30)	36.40 (32.90–39.40）	35.55 (31.40–39.30）	0.019
Serum calcium (mmol/L)	2.01 (1.82–2.16)	1.96 (1.78–2.12)	2.05 (1.88–2.20)	0.255
Serum phosphorus (mmol/L)	1.82 (1.50–2.12)	1.86 (1.53–2.14)	1.76 (1.46–2.11)	0.006

*Note*: normal reference range: serum albumin: 40.00–55.00g/L; serum calcium: 2.08–2.80 mmol/L; serum phosphorus: 0.83–1.96 mmol/L. RRF was calculated as (urine creatinine [μmol/l] × urine volume [ml])/(plasma creatinine [μmol/l] × minutes) and corrected for body surface area.

PLT: platelet counts; BMI: body mass index; RRF: residual renal function.

### Risk factors for higher incidence of new-onset CV events

The significant risk factors for the higher incidence of new-onset CVD events in PD patients are given in Table 3 by adjusting for relevant covariates. A higher incidence of new-onset CVD events was associated with age and high PDW levels (Table 3).

**Table 3.** Risk factors for new-onset CVD.

Risk factors	Univariable Cox regression		Multivariable Cox regression	
	HR (95%CI)	*p*-Value	HR (95%CI)	*p*-Value
PDW (>13.2 fL vs.≤13.2 fL)	1.695 (1.164–2.468)	0.006	1.736 (1.173–2.567)	0.006
PLT ( >163.00 (10^9^/L) vs.≤163.00 (10^9^/L))	0.887 (0.613–1.282)	0.522		
Age (per 1 year increase)	1.020 (1.007–1.034)	0.003	1.022 (1.005–1.038)	0.009
History of diabetes (yes vs. no)	1.719 (1.109–2.666)	0.015		
History of hypertension (yes vs. no)	1.079 (0.711–1.637)	0.721		
Lymphocyte (per 1 × 10^9^/L increase )	0.892 (0.584–1.362)	0.597		
RRF (per 1 ml/min/1.73 m^2^ increase)	0.951 (0.869–1.040)	0.268		
Total cholesterol (per 1 mmol/L increase)	0.972 (0.827–1.143)	0.734		
Total triglycerides (per 1 mmol/L increase)	0.974 (0.790–1.201)	0.808		
Serum urea nitrogen (per 1 mmol/L increase)	0.990 (0.970–1.010)	0.321		
Uric acid (per 1 μmol/L increase)	0.999 (0.998–1.001)	0.273		
Serum creatinine (per 1 μmol/L increase)	1.000 (0.999–1.001)	0.896		
Serum calcium (per 1 mmol/L increase)	0.814 (0.429–1.545)	0.530		
Serum phosphorus (per 1 mmol/L increase)	0.767 (0.530–1.110)	0.160		

CVD: cardiovascular disease; PLT: platelet; PDW: platelet distribution width; RRF: residual renal function.

### *PLT indices and new-onset CVD eve*nts

**Figure 4.**
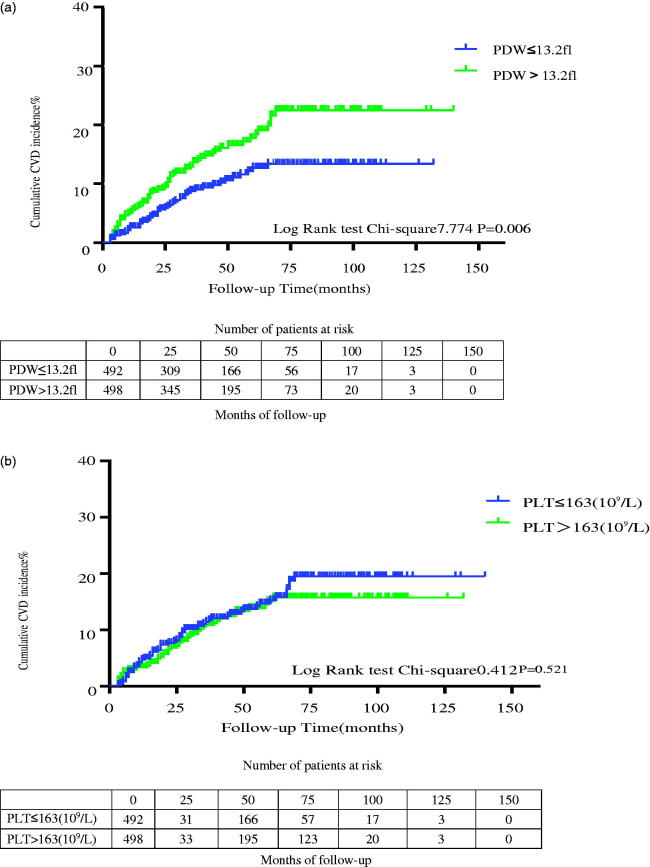
(a) Crude analyses of new-onset cardiovascular disease of PDW with Kaplan–Meier estimates. (b) Crude analyses of new-onset cardiovascular disease of platelet with Kaplan–Meier estimates. PLT: platelet counts; PDW: platelet distribution width.

Kaplan–Meier survival curves for patients with different groups are shown in Figure 4. In crude analysis, the Kaplan–Meier survival curve showed that there was a significant difference in new-onset CVD between the two PDW groups (*p* = 0.006) (Figure 4(a)). There was no significant difference between groups of PLT and their association with new-onset CVD (Figure 4(b)).

The hazard ratios (HRs) of PDW associated with new-onset CVD from adjusted Cox proportional hazards models are listed in Table 4. Regardless of the adjustment model used, the high PDW group was associated significantly with a higher incidence of new-onset CVD events compared with the low PDW group (HR = 1.784 95%CI 1.173–2.712, *p* = 0.007, HR= 1.769 95%CI 1.161–2.693, *p* = 0.008 and HR = 1.862 95%CI 1.205–2.877, *p* = 0.005).

**Table 4.** Relationship between PDW and new-onset CVD.

	PD*W* > 13.2fL HR (95%CI)	*p*-Value
Unadjusted	1.695 (1.164–2.468)	0.006
Model 1	1.784 (1.173–2.712)	0.007
Model 2	1.769 (1.161–2.693)	0.008
Model 3	1.862 (1.205–2.877)	0.005

With the PDW ≤13.2 fL group as reference.

Model 1: Sex, age, body mass index.

Model 2: Model 1 plus history of diabetes, and history of hypertension.

Model 3: Model 2 plus lymphocyte, total cholesterol, total triglycerides, serum urea nitrogen, uric acid, serum creatinine, serum calcium, and serum phosphorus.

CVD: cardiovascular disease; PDW: platelet distribution width; HR: hazard ratio.

### ROC curves method for platelet indices on CVD risk

**Figure 5.**
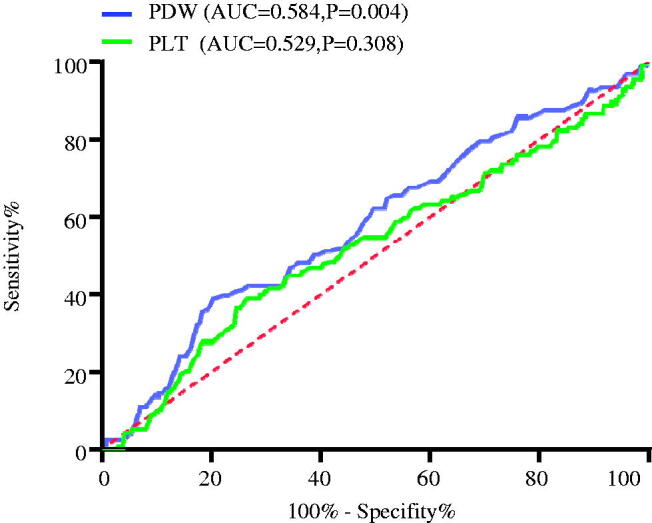
ROC curves of PDW and PLT. PDW: platelet distribution width; PLT: platelet counts.

To assess the predictive accuracy of the two platelet indices on CVD events, ROC curves were applied (Figure 5). It was shown that the AUC of PDW predicting CVD events was greater than 0.5 (AUC = 0.584, *p* = 0.004), but the AUC of PLT predicting CVD events failed to attach statistical significance (AUC = 0.529, *p* = 0.308). These results revealed that PDW was one of the parameters influencing CVD events in patients undergoing PD. In contrast, PLT was not associated with CVD events in PD patients.

**Figure 6.**
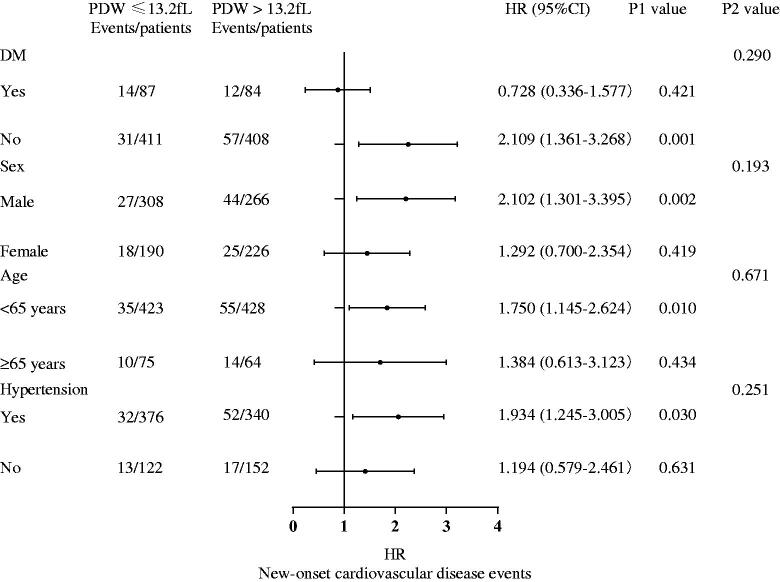
The results of subgroup analysis with the forest plot. DM: diabetic mellitus; PDW: platelet distribution width; HR: hazard ratio; P1 value: P for each subgroup; P2 value: P for interaction.

### PDW and the new-onset CVD events in different groups

We performed subgroup analysis in some subgroups including sex, age, history of hypertension, and history of diabetes. We explored the interaction between the subgroups and PDW. The forest plot showed no interaction between the subgroups (Figure 6).

## Discussion

The results reveal that PDW is associated with the incidence of new-onset CVD in PD patients, but PLT is not associated with new-onset CVD. Moreover, PDW is the basic test index of blood routine. These findings are beneficial for clinical practice because of the widespread use and economic benefits of blood routine.

PDW is a special marker of platelet activity [31]. High platelet activity means that platelets have a greater ability to promote inflammation and coagulation, which is closely associated with CVD events [32].

In previous studies, there have been many studies to prove that elevated PDW is related to the occurrence of CVD events. In one study, PLT and PDW are independently associated with CVD events in CKD patients without dialysis [29]. Another study suggests that pre-procedural PDW may be an independent predictor of both in-hospital and long-term adverse [33]. Besides this, PDW seems to be an independent marker of STEMI in young patients [34]. Our study is consistent with these findings, which further proves the possibility that elevated PDW is associated with CVD events in PD patients. However, in a recent retrospective study, PDW is related to heart failure in the general population, despite there might be a threshold in PDW level [24]. One explanation may be that patients with PD are a kind of population with more active systemic inflammation and worse systemic nutrition [35], and the level of PDW is closely related to the inflammatory state [36].

On the contrary, the association between PLT and CVD events has been reported on studies. Vidwan et.al. reported that baseline PLT was a strong and independent risk factor for bleeding and vascular complications in patients undergoing coronary angiography [37]. Through a population-based cohort study, Vinholt et al. found that high PLT was associated with mortality, future cardiovascular disease, and future cancer [38]. However, our study failed to attach these results. PD patients often received a combination of medications. We suggest that the use of vitamin D and erythropoietin in PD patients will affect PLT through affecting bone marrow hematopoietic function [39,40]. Thus, PDW may be more stable than PLT in PD patients.

In many studies, diabetes has been shown as an independent risk factor for cardiovascular events [41,42]. Hyperglycemia adversely affects vascular structure and function through multiple mechanisms, including oxidative stress, inflammation, and procoagulant activity [43]. In our study, we found that PDW had a greater predictive value for CVD events in PD patients without diabetes. This may be because the pro-inflammatory and pro-thrombotic effects of diabetic mellitus are likely to weaken the effects of PDW on CVD events in PD patients.

Our study has several limitations. Our study was divided into two groups, which could only prove that high PDW was associated with new CVD in PD patients, but there was no evidence that there was a threshold in PDW level for identifying high-risk groups for CVD events in PD patients. We excluded glucocorticoids and immunosuppressants. However, due to lack of data, other drugs like antithrombotic agents were not excluded, which might lead to bias in our results. Whether other drugs affect platelet index needed further study. At last, due to a lack of data, neither relevant platelet indices including plateletcrit, mean platelet volume, and platelet large cell ratio, nor platelet variables such as platelet production cytokines, immature platelet counts, immature platelet fraction, and highly fluorescent immature platelet fraction were involved. Thus, a more comprehensive analysis of platelet index and new-onset CVD in PD patients is not available.

## Conclusion

In conclusion, the higher levels of PDW were associated with the incidence of new-onset CVD in PD patients, whereas PLT was not associated with new-onset CVD in PD patients.

## Data Availability

The datasets in this study are available from the corresponding author on reasonable request.
